# Inhibiting toll-like receptor 4 signaling ameliorates pulmonary fibrosis during acute lung injury induced by lipopolysaccharide: an experimental study

**DOI:** 10.1186/1465-9921-10-126

**Published:** 2009-12-18

**Authors:** ZhengYu He, YeSen Zhu, Hong Jiang

**Affiliations:** 1 Department of Anesthesiology, Ninth People's Hospital, Shanghai Jiao Tong University School of Medicine, Shanghai 200011, China

## Abstract

**Background:**

Toll-like receptor 4 (TLR4) is essential in lipopolysaccharide (LPS)-induced fibroblast activation and collagen secretion in vitro. However, its effects on the process of lung fibroblast activation and fibrosis initiation during LPS induced acute lung injury (ALI) remain unknown. The goal of the present study was to determine the effect of inhibiting TLR4 on LPS-induced ALI and fibrosis in vivo.

**Methods:**

The ALI model was established by intraperitoneal injection of LPS in mice. TLR4-small hairpin RNA (shRNA) lentivirus was injected intravenously into the mice to inhibit TLR4 expression. mRNA and protein levels were detected by real-time PCR and Western-blot analysis, respectively. The contents of the C-terminal propeptide of type I procollagen (PICP) in bronchoalveolar lavage fluid (BALF) were detected by ELISA, and the degree of fibrosis was detected by van Gieson collagen staining, the hydroxyproline assay, and alpha smooth muscle actin (α-SMA) immunohistochemical staining.

**Results:**

Overexpression of TLR4, type I procollagen, alpha-SMA, and p-AKT in murine pulmonary tissue after intraperitoneal injection of LPS at 72 hours and 28 days were detected. Moreover, the degree of fibrosis was shown to increase by ELISA analysis of PICP in BALF, van Gieson collagen staining, the hydroxyproline assay, and α-SMA immunohistochemical staining. All of these changes were alleviated by intravenous infection with TLR4-shRNA lentivirus.

**Conclusions:**

Inhibiting TLR4 signaling could ameliorate fibrosis at the early stage of ALI induced by LPS.

## Background

Lipopolysaccharides (LPSs), which constitute the principal endotoxin of Gram-negative bacteria, are important factors in acute lung injury (ALI). Although ALI traditionally was considered to progress through successive stages of exudation, proliferation, and fibrosis, The process of fibrosis recently was found to be initiated during the early stage of ALI[1]. Toll-like receptor 4 (TLR4) is a widely distributed LPS receptor in lung tissue. The role of TLR4 in infectious and even non-infectious induced fibrosis has aroused increasing concern[2,3].

LPSs exert their biological effects through TLR4. LPSs promote TLR4 expression in the mononuclear phagocytic system and promote the secretion of pro-fibrotic cytokines, including transforming growth factor-β1 (TGF-β1). Through a series of direct and indirect pathways, LPSs stimulate lung fibroblast proliferation and alpha smooth muscle actin (α-SMA) expression and differentiate lung fibroblasts into myofibroblasts to synthesize and secrete collagen. The carboxyterminal propeptide of type I procollagen (PICP) is a marker of type I collagen synthesis and is closely related to ALI prognosis[4]. LPS stimulation results in the deposition of extracellular matrix (ECM) and eventually diffuses interstitial pulmonary fibrosis[5]. LPSs, by inducing the paracrine action of some inflammatory factors, induce lung fibroblast chemotaxis, activate pulmonary macrophages, and work together with other cells during the pulmonary fibrosis process[6,7]. TLR4 also is expressed in lung fibroblasts. Our preliminary studies illustrated that LPSs could activate the phosphoinositide3-kinase-Akt (PI3K-Akt) pathway via TLR4 in primary cultured mouse lung fibroblasts, which subsequently promoted the synthesis of α-SMA and the activation of fibroblasts, thereby directly promoting collagen secretion and accelerating the process of pulmonary fibrosis through the upregulation of integrin β1 expression[8].

Because TLR4 is a key point in the first step of the LPS signal transduction pathway, inhibiting TLR4 expression and the subsequent blocking of its signal pathway may represent an effective way to ameliorate the acute inflammatory reaction and pulmonary fibrosis that occur during ALI. In vitro research found that inhibiting TLR4 can alleviate the LPS-induced inflammatory reaction during the early stages of ALI[9]. However, the effects of inhibiting TLR4 expression at the tissue level on the process of lung fibroblast activation and fibrosis initiation remain unknown.

In this study, we used the lentivirus-mediated RNA interference technique to inhibit TLR4 gene expression in mouse lung tissue in order to observe lung fibroblasts activation, pulmonary fibrosis initiation, and progression during LPS-induced ALI. We also investigated the role that inhibiting TLR4 plays in ameliorating LPS-induced ALI and fibrosis.

## Methods

### Animals

The experiment was performed using 8-week-old male C57BL/6 mice obtained from the Shanghai SLAC Laboratory Animal Co. Ltd. They were allowed free access to water and commercial rodent chow and were housed in pathogen-free cages. This study was approved by Shanghai Jiaotong University School of Medicine Animal Care and Use Committee. All procedures were performed in accordance with the guidelines of the National Institutes of Health for animal care[10].

### Small hairpin RNA (shRNA) lentivirus vector of the TLR4 gene

pGCL-GFP-lentivirus expressing small hairpin RNAs (shRNAs) targeting the TLR4 gene (hereafter called TLR4-shRNA lentivirus) and negative control lentivirus expressing non-targeting sequence were bought from Shanghai Genechem Co. Ltd. (Shanghai, China).

Forty-eight hours after infecting mice with TLR4-shRNA lentivirus via intravenous injection, TLR4-shRNA lentivirus could infect both parenchymal and marrow-derived cells in mice lung tissue with high efficiency. It could integrate into the genome of host cells and exhibit stable expression for more than 28 days.

### Experimental design

Intraperitoneal injection of LPSs in mice can establish a model of acute lung injury[11]. In this study, 60 mice were randomly divided into five groups (n = 12):

• Blank control group (Group BC): Physiological saline was administered intraperitoneally without lentivirus infection or LPS stimulation.

• Negative control group (Group NC): These animals were infected with negative control shRNA lentivirus, without LPS stimulation.

• Positive control group (Group PC): These animals were infected with negative control shRNA lentivirus and stimulated with LPS.

• TLR4 inhibition group (Group TI): Animals in this group were infected with TLR4-shRNA lentivirus without LPS stimulation.

• TLR4 inhibition group stimulated with LPS (Group TI+L): Animals in this group were infected with TLR4-shRNA lentivirus and then stimulated with LPS.

In groups TI and TI+L, 5 × 10^7 ^TU/ml of TLR4-shRNA lentivirus were injected intravenously into the mice to inhibit TLR4 mRNA expression; the same dose of negative control shRNA lentivirus was applied in groups NC and PC. In groups PC and TI+L, 5 mg/kg LPS (serotype O55: B5; Sigma Chemical, Taufkirchen, Germany) from *Escherichia coli *were injected intraperitoneally for 3 consecutive days 48 hours after lentivirus infection to induce endotoxemia and pulmonary fibrosis. The same volume of physiological saline was injected in the same way in group BC, NC, and TI to serve as a control.

After LPS (or physiological saline) administration, each group was subdivided into two subgroups labeled "3d" (72 hours after LPS or physiological saline administration) and "4w" (4 weeks after LPS or physiological saline administration). Thus, the experiment consisted of 10 groups, each containing 6 mice. The mice were monitored on a daily basis for 72 hours (early period of ALI) or 28 days (later period of ALI) after LPS administration. At the appropriate time, the mice were euthanized with intraperitoneal injection of pentobarbital (120 mg/kg), followed by exsanguination by closed intracardiac puncture. Lung tissues were quickly removed and processed as described below.

### Bronchoalveolar lavage (BAL) and measurement of PICP by ELISA

BAL was conducted for the measurement of PICP in bronchoalveolar lavage fluid (BALF) as described elsewhere[12]. BAL fluid was centrifuged, and the supernatant was used for the measurement of PICP by ELISA[4].

### Histopathological examination

Lung tissue excised 72 hours or 28 days after LPS application was subjected to hematoxylin-eosin (HE) and van Gieson (VG) staining to detect inflammation or collagen deposition during ALI. The severity of fibrosis was assessed semiquantitatively using the Ashcroft score: Grade 0, normal lung; Grade 1, minimal fibrous thickening of alveolar or bronchiolar walls; Grade 3, moderate thickening of walls without obvious damage to the lung architecture; Grade 5, increased fibrous with definite damage to the lung architecture and formation of fibrous bands or small fibrous masses; Grade 7, severe distortion of the architecture and a large fibrous area; Grade 8, total fibrous obliteration of the field[13]. All of the sections were evaluated and scored independently by two investigators in a blinded manner. Severity of fibrotic changes in each lung section was assessed as the mean score for severity from the observed microscopic fields.

### Hydroxyproline assay in lung tissue

Pulmonary collagen secretion and deposition 28 days after application of LPS in mice were quantitatively analyzed using the hydroxyproline assay. The samples were hydrolyzed with 6 N HCl at 110°C for 18 hours following Woessner's method[14]. After neutralization with NaOH, the hydrolyzates were diluted with distilled water. Hydroxyproline in the hydrolyzates was assessed colorimetrically at 550 nm for the presence of p-dimethylaminobenzaldehyde. Results were expressed as micrograms of hydroxyproline per lung.

### Real-time PCR

The mRNA expression levels of type I procollagen, TLR4, integrin β1, and α-SMA were analyzed using a real-time PCR quantification method. Housekeeping gene actin was used as a control. Total RNA was isolated from tissue with the RNeasy kit (Trizol, Invitrogen, USA) and then reverse-transcribed into cDNA with a reverse transcription kit (M-MLV, Promega, USA). Sequence-specific primers were designed as follows: Actin-F: 5'-TGCGTGACATCAAAGAGAAGC-3', Actin-R: 5'-CAGCACTGTGTTGGCATAGAG-3'; TLR4-F: 5'-ATGGCATGGCTTACACCACC-3', TLR4-R: 5'-GAGGCCAATTTTGTCTCCACA-3'; integrin β1-F: 5'-TAAACCTCTGGGCTTCACTG-3', integrin β1-R: 5'-TGTCTTCACTGTTCACTTCATC-3'; α-SMA-F: 5'-CTGCCGAGCGTGAGATTG-3', α-SMA-R: 5'-ATAGGTGGTTTCGTGGATGC-3'; type I procollagen-F: 5'-TGAGACAGGCGAACAAGG-3', type I procollagen-R: 5'-CAGGAGAACCAGCAGAGC-3'. Real-time PCR was performed on the IQ5 PCR System (Bio-Rad, USA) with an initial denaturing step at 95°C for 15 seconds, 45 denaturing cycles at 95°C for 5 seconds, and an annealing step at 60°C for 30 seconds. Relative expression of real-time PCR products was determined by using the ΔΔCt method[15] to compare target and housekeeping gene expression.

### Western blot analysis

Protein levels of TLR4, integrin β1, α-SMA, and Akt phosphorylation products (p-AKT) in each group were detected by Western blot analysis. Tissues were collected and lysed with protease inhibitor in 1 × lysis buffer on ice for 10 to 15 minutes. Samples were centrifuged and supernatants were collected. Protein quantification was performed using the BCA method, and SDS-PAGE electrophoresis was performed. Proteins were transferred to PVDF membranes and detected using the ECL+plusTM Western blotting system kit (Amersham, USA). Primary antibody (TLR4 from Santa Cruz, p-Akt from CST, α-SMA and integrin β1 from Abcam) was diluted to 1:1000 in this experiment. Data were analyzed by gel imaging systems and corrected using β-actin as the internal control.

### Immunohistochemistry

To determine α-SMA expression in lung tissue, paraffin-embedded tissue was processed using the avidin-biotin immunoperoxidase method. The antibody against α-SMA (Abcam, 1:100) was used for immunohistochemistry. All of the sections were evaluated and scored independently by two investigators in a blinded manner.

### Statistical methods

All data were represented as mean ± standard deviation, and SPSS12.0 software (SPSS, Inc., USA) was used for statistical analysis. The homogeneity of variance data were analyzed with the one-way analysis of variance LSD test and the heterogeneity of variance data were analyzed with the Kruskal Wallis rank sum test. Statistical significance was defined at P < 0.05.

## Results

### TLR4-mediated pulmonary fibrosis induced by LPS

**Figure 1:**
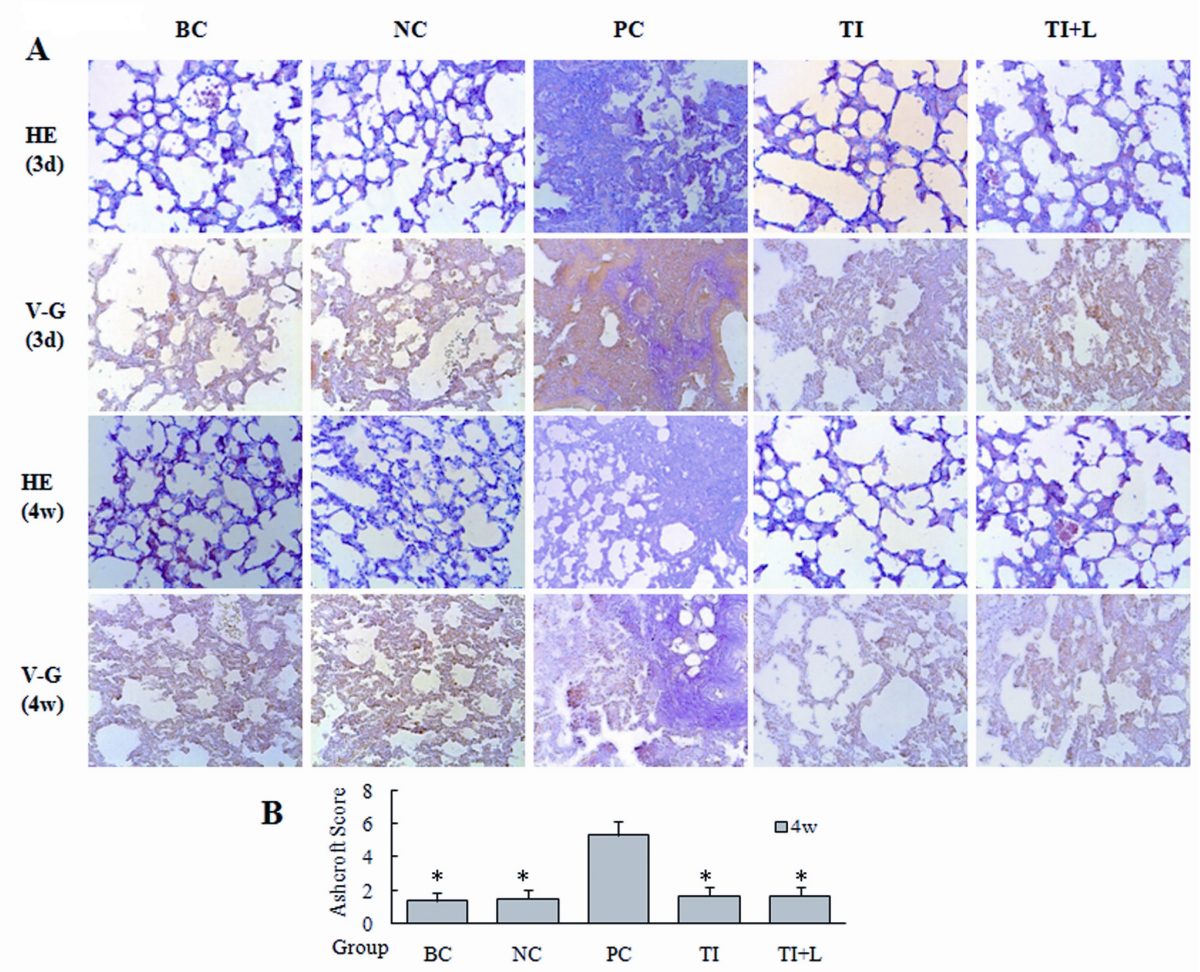
**Inflammation and fibrosis in mouse lung tissue after LPS challenge**. (A) The pathological changes in mouse lung tissue 72 hours and 28 days after intraperitoneal injection of LPS were observed by means of HE staining (HE, A, magnification ×200). Pulmonary fibrosis was observed by means of Van-Gieson staining (VG, magnification ×200). (B) The Ashcroft fibrosis score was used to compare the degrees of the pulmonary fibrosis 28 days after application of LPS in mice. The mice showed obvious inflammatory reactions in lung tissue 72 hours after LPS challenge. Typical pulmonary interstitial fibrosis appeared 4 weeks later. Infection with TLR4-shRNA lentivirus significantly inhibited the inflammatory reaction and fibrosis induced by LPS. BC: blank control group; NC: negative control group; PC: positive control group; TI: TLR4 inhibition group; TI+L: TLR4 inhibition group stimulated with LPS; 3d: specimens were collected 72 hours after LPS (or physiological saline) challenge. 4w: specimens were collected 28 days after LPS (or physiological saline) challenge. Each subgroup contains 6 specimens respectively (n = 6). Results were expressed as mean ± standard deviation indicated with column graph and error bar. Statistical significance was defined at p values < 0.05. *: P < 0.05 versus PC.

First, we determined the role of TLR4 in the activation of lung fibroblasts and collagen secretion at the tissue level. As shown in Figure 1, HE and VG staining of lung tissue stimulated with LPS revealed that 72 hours after the LPS challenge, the lung tissue showed obvious inflammatory reactions, and typical interstitial fibrosis occurred 28 days after LPS challenge. At the same time, the Ashcroft score increased significantly compared with the blank and negative control groups (P < 0.05).

**Figure 2:**
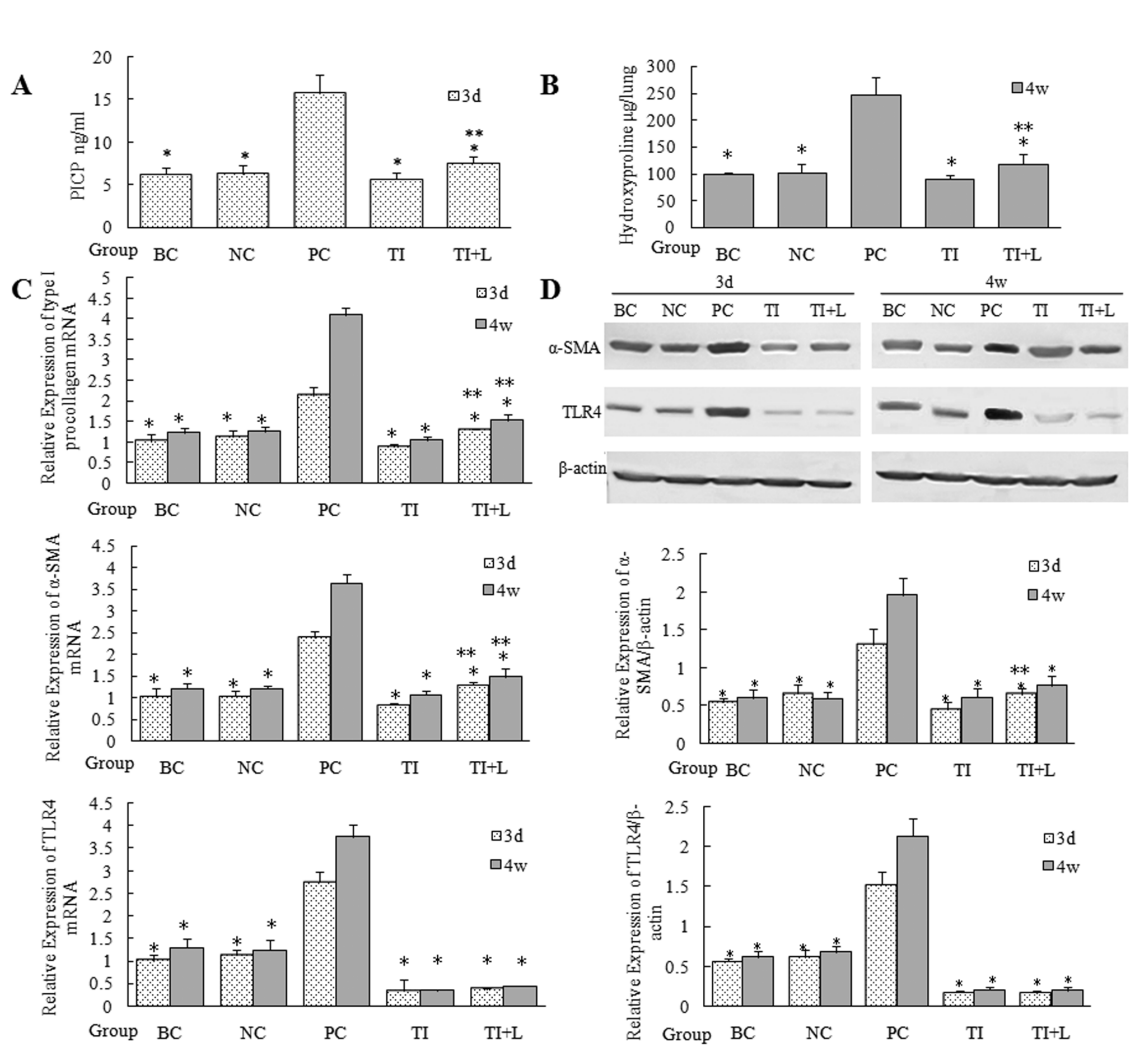
**Collagen synthesis and secretion in mouse lung tissue after LPS challenge**. (A) ELISA was used to detect PICP contents in mouse BALF to compare the synthesis of pulmonary type I collagen 72 hours after intraperitoneal injection of LPS. (B) Pulmonary collagen secretion and deposition 28 days after application of LPS in mice were quantitatively analyzed with hydroxyproline assay. (C) The mRNA expression of type I procollagen, α-SMA, and TLR4 in mouse lung tissue was detected by real-time PCR. (D) Protein expression of α-SMA and TLR4 were detected using Western blots. Type I procollagen, α-SMA, and TLR4 expression in mouse lung tissue increased significantly 72 hours after LPS challenge and become intensive 4 weeks later. Infection with TLR4-shRNA lentivirus significantly inhibited these changes. BC: blank control group; NC: negative control group; PC: positive control group; TI: TLR4 inhibition group; TI+L: TLR4 inhibition group stimulated with LPS; 3d: specimens were collected 72 hours after LPS (or physiological saline) challenge. 4w: specimens were collected 28 days after LPS (or physiological saline) challenge. Each subgroup contains 6 specimens respectively (n = 6). Results were expressed as mean ± standard deviation indicated with column graph and error bar. Blots were representative of six separate experiments. Statistical significance was defined at p values < 0.05. *: P < 0.05 versus PC; **:P < 0.05 versus TI.

**Figure 3:**
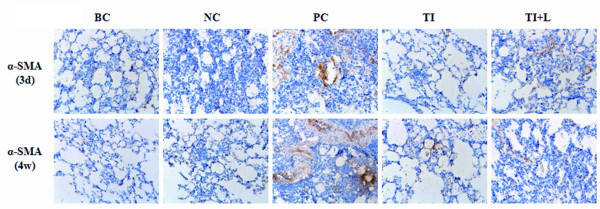
**Expression of α-SMA in mouse lung tissue after LPS challenge**. Paraffin-embedded mouse lung tissue samples were deparaffinized, rehydrated, and processed to detect α-SMA using the avidin-biotin immunoperoxidase method. Representative images (×200) of samples are shown. BC: blank control group; NC: negative control group; PC: positive control group; TI: TLR4 inhibition group; TI+L: TLR4 inhibition group stimulated with LPS; 3d: specimens were collected 72 hours after LPS (or physiological saline) challenge. 4w: specimens were collected 28 days after LPS (or physiological saline) challenge. Each subgroup contains 6 specimens respectively (n = 6).

PICP is a segment of type I procollagen degraded from the C-terminal by the procollagen C-endopeptidase, and it is closely related to ALI prognosis[4]. It has been investigated in a number of lung diseases as a marker of type I collagen synthesis[16]. The ELISA results showed that the content of PICP in mouse BALF increased significantly 72 hours after LPS challenge (i.e., at the early stage of ALI) (Figure 2A). Furthermore, at the late stage of ALI (28 days after LPS challenge), the hydroxyproline assay demonstrated that the content of hydroxyproline, a marker of collagen synthesis, in mouse lung tissue was significantly higher than that in the blank and the negative control groups (P < 0.05) (Figure 2B). Real-time PCR and Western blot analyses showed that upregulation of type I procollagen and α-SMA, a marker of fibroblast activation, appeared 72 hours after LPS challenge and became intensive 4 weeks later (Figure 2C, 2D). Immunohistochemical staining for α-SMA also illustrated α-SMA upregulation in the PC group (Figure 3).

The expression of TLR4 in lung tissue also increased significantly 72 hours after LPS challenge, as detected by real-time PCR and Western blot analysis (P < 0.05) (Figure 2C, 2D). However, infection with TLR4-shRNA lentivirus inhibited TLR4 expression at the mRNA and protein levels (Figure 2C, 2D, Figure 3), downregulated the content of PICP in mouse BALF (Figure 2A) and hydroxyproline (Figure 2B), inhibited the expression of α-SMA and type I procollagen (Figure 2C, 2D, Figure 3), and therefore alleviated the degree of pulmonary fibrosis induced by LPS (Figure 1). However, TLR4-shRNA lentivirus treatment could not completely inhibit PICP (Figure 2A), hydroxyproline (Figure 2B), α-SMA, and type I procollagen (Figure 2C, 2D, 3) expression, as shown by the comparison between the TI and TI+L groups.

### LPS activates the PI3K-Akt pathway via TLR4 and upregulated integrin β1 expression

**Figure 4:**
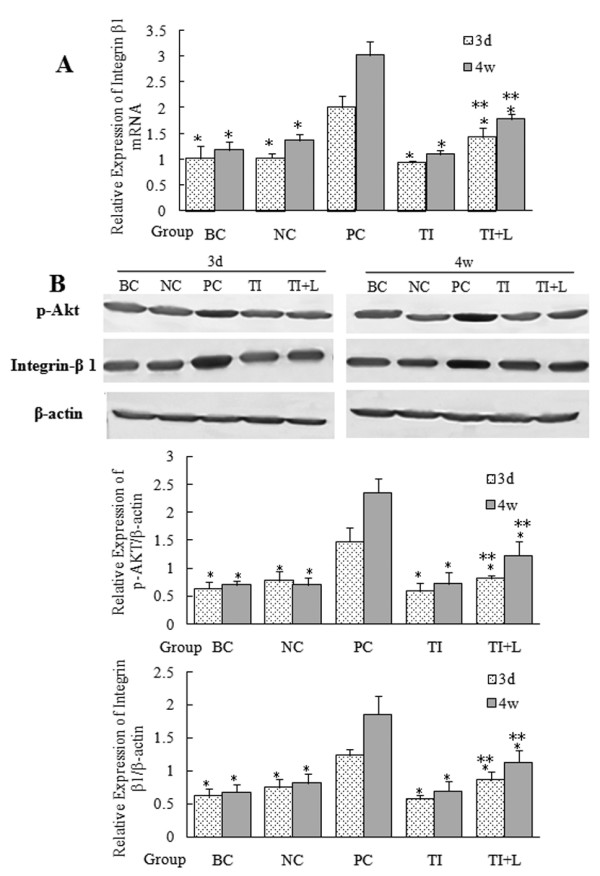
**Activation of the PI3K-Akt pathway and expression of integrin β1 in mouse lung tissue after LPS challenge**. The p-AKT or integrin β1 in mouse lung tissue was detected by real-time PCR (A) or Western blot (B). P-Akt, which reflected Akt phosphorylation levels, and integrin β1 expression increased significantly 72 hours after LPS challenge and become intensive 4 weeks later. Infection with TLR4-shRNA lentivirus significantly inhibited these changes. BC: blank control group; NC: negative control group; PC: positive control group; TI: TLR4 inhibition group; TI+L: TLR4 inhibition group stimulated with LPS; 3d: specimens were collected 72 hours after LPS (or physiological saline) challenge. 4w: specimens were collected 28 days after LPS (or physiological saline) challenge. Each subgroup contains 6 specimens respectively (n = 6). Results were expressed as mean ± standard deviation indicated with column graph and error bar. Blots were representative of six separate experiments. Statistical significance was defined at p values < 0.05. *: P < 0.05 versus PC; **:P < 0.05 versus TI.

Our preliminary studies showed that LPS could activate the PI3K-Akt pathway via TLR4 in primary cultured mouse lung fibroblasts. Herein, we examined the molecular mechanism of this pathway at the tissue level. Real-time PCR and Western blot analysis showed that the Akt phosphorylation levels, which indicate the activation of the PI3K-Akt pathway, and integrin β1 expression were significantly increased 72 hours after LPS challenge and became intensive 4 weeks later compared with the blank and negative control groups (P < 0.05) (Figure 4). These changes could be inhibited by infection with the TLR4-shRNA lentivirus, but TLR4-shRNA lentivirus could not completely inhibit p-AKT and integrin β1 expression.

## Discussion

Although it has long been known that an inflammatory reaction accompanies fibrosis diseases[17], the mechanism that underlies the interaction of the two processes remains unclear. Past reports of TLR4 expression in lung tissue after LPS challenge have been controversial [2,18-21], possibly because of differing experimental designs and because organisms can initiate different regulatory mechanisms depending on physiopathologic state [19,22-24]. Our experimental results showed that the expression of TLR4 in mice lung tissue increased consistently 3 days and 28 days after LPS challenge, which was consistent with data from some previous reports [2,20,21,24]. These results suggest that some positive feedback mechanism, such as crosstalk between different intracellular signal transduction pathways of various receptors, may exist and continue to affect the expression of TLR4. Furthermore, as the overexpression of TLR4 in lung tissue was accompanied by the aggravation of fibrosis, it is reasonable, based on our previous in vitro study, to speculate that the LPS challenge could induce the overexpression of TLR4 in mice lung fibroblasts, thereby enhancing their sensitivity to LPS and activating lung fibroblasts to initiate the progress of pulmonary fibrosis.

α-SMA is a marker of fibroblast activation and its presence indicates the occurrence of fibroblast transition towards myofibroblast. Myofibroblasts can synthesize and secrete collagen to the ECM and thereby lead to pulmonary interstitial fibrosis. PICP is a segment of type I procollagen that is degraded from the C-terminal by the procollagen C-endopeptidase, and it is closely related to ALI prognosis[4]. It has been investigated in a number of lung diseases as a marker of type I collagen synthesis[16]. Our results illustrated the positive effects of TLR4 inhibition in preventing the inflammatory reaction and fibrosis in ALI induced by LPS in vitro[8]. In this experiment, the expression of TLR4 in mouse lung tissue increased significantly 72 hours after LPS treatment and was accompanied by increased expression of α-SMA, type I procollagen, and PICP; moreover, the degree of pulmonary fibrosis became intense 4 weeks after LPS challenge (Figures 1, 2, 3). This result indicates that LPS can upregulate TLR4 expression and activate lung fibroblasts after TLR4 activation at the early inflammatory reaction stage of ALI, thereby promoting collagen synthesis and secretion and causing pulmonary interstitial fibrosis. Macrophage recruitment and the proinflammatory response to *Mycobacterium tuberculosis *are impaired in TLR4 mutant mice, resulting in chronic infection and impaired elimination of mycobacteria. Therefore, TLR4 signaling is required to mount a protective response during chronic infection[25]. Because genetic mutation of TLR4 resulted in impairment of the development of protective immunity, in the present study we used the lentiviral shRNA infection approach to specifically reduce TLR4 expression in mice and studied the effects of suppression of TLR4 expression on lung tissue after LPS challenge. This approach overcomes the shortcomings of traditional plasmid transfection, in which the transfection efficiency for the primary culture cells is too low and the duration of expression in the host is too short. Therefore, this technique may be a useful gene therapy approach for treating pulmonary fibrosis during ALI. TLR4-shRNA lentivirus infection may inhibit the expression of α-SMA and type I procollagen (Figure 2C, 2D, 3) and therefore alleviate the degrees of pulmonary fibrosis induced by LPS (Figure 1).

Integrin exerts a dual regulatory function to accelerate the inflammatory reaction and the development of pulmonary interstitial fibrosis[26,27]. PI3K is a converging point for multiple signal transduction pathways and can be activated by different signals, including integrins, TGF-β1, and insulin-like growth factor-1 (IGF-1)[26,28,29]. The PI3K-Akt pathway is involved in α-SMA expression,[30,31] TGF-β-mediated cytokine release, and upregulation of integrin expression,[29,32] thereby playing a critical role in the development of fibrosis diseases. Our previous in vitro study showed that the PI3K-Akt pathway could promote LPS-induced fibroblast activation and collagen secretion as part of the TLR4-mediated signal transduction pathway in lung fibroblast[8]. In this experiment, mouse lung tissue showed increased integrin β1 expression and Akt phosphorylation levels 72 hours after LPS challenge. However, infection with TLR4-shRNA lentivirus could inhibit both the LPS-induced upregulation of integrin β1 and Akt phosphorylation, suggesting that LPS plays these roles through the PI3K-AKT pathway via TLR4 (Figure 4). It would be more clinically relevant if the PI3k/AKT pathway had been inhibited pharmacologically in animals with an intact TLR4 receptor; such a finding would prove its importance in the development of fibrosis. This experiment will be conducted in the future.

Although our work and previous studies have shown the positive effects of TLR4 inhibition in preventing the inflammatory reaction and fibrosis in ALI induced by endotoxins of a Gram-negative bacillus,[9] it was also reported in non-Gram-negative bacteria[22,25,33] or even non-infectious factors[34] induced ALI animal models, the absence or inhibition of TLR4 expression could lead to aggravation of inflammatory injury and increased animal mortality. This suggests that as a pathogen pattern recognition receptor, TLR4 has a protective effect in regulating cellular immune function, maintaining the stability of alveolar epithelium, and reconstruction after inflammatory reaction. However, excessive expression and activation of TLR4 in certain pathological states may result in uncontrolled inflammatory responses and fibrosis. Therefore, the regulatory mechanisms of TLR4 expression in different pathological states and the means to prevent the overexpression of TLR4 are important directions for further study.

Infection with TLR4-shRNA lentivirus could not completely inhibit PICP (Figure 2A), hydroxyproline (Figure 2B), α-SMA, and type I procollagen (Figure 2C, 2D, Figure 3) expression and also could not completely inhibit p-AKT and integrin β1 expression (Figure 4) (as shown by the comparison between the TI and TI+L group). Thus, fibrosis induced by LPS is not completely blocked by TLR4 inhibition. This result suggests that TLR4 is not the only activator of the PI3K-Akt pathway and the resulting fibrosis. The residual protein expression may represent the activation of different pathways. Activators of the PI3K-Akt pathway, such as integrins, TGF-β1, and IGF-1[26,28,29], should be studied further.

## Conclusions

In this experiment, we used an in vivo experiment to show that TLR4 could activate the PI3K-Akt pathway and upregulate expression of integrin β1, thereby promoting fibroblast activation and collagen secretion and initiating pulmonary fibrosis at the early stage of ALI. Inhibiting TLR4 expression by transfection with the TLR4-shRNA lentivirus inhibited the process of fibrosis at the early stage of ALI. Thus, this treatment might decrease the severity of LPS-induced ALI and pulmonary fibrosis and subsequently improve the prognosis of the disease.

## Competing interests

The authors declare that they have no competing interests.

## Authors' contributions

ZhengYu He performed the experimental studies and drafted the manuscript. YeSen Zhu designed and planned the experiments and assisted with several phases of the study. Hong Jiang designed the experimental set up, supervised the experimental work, participated in the manuscript preparation and contributed important intellectual content. All authors have read and approved the final manuscript.
